# Continuous positive airway pressure ventilation during whole lung lavage for treatment of alveolar proteinosis -A case report and review of literature

**DOI:** 10.4103/1658-354X.76475

**Published:** 2011

**Authors:** Abdelazeem El-Dawlatly, Waseem Hajjar, Sami Alnassar, Reem Alsafar, Ahmed Abodonya

**Affiliations:** *College of Medicine, King Saud University, Riyadh, KSA*

**Keywords:** *Lactic acidosis*, *toluene*, *hyperlactemia*

## Abstract

Pulmonary alveolar proteinosis (PAP) is a rare disease that affects young population usually in the age group of 20-40 years, characterized by the deposition of lipoproteinacious material in the alveoli secondary to abnormal processing of surfactant by macrophages. We report a case of a 15-year-old female who had history of cough with sputum for 3 days along with fever. She was seen in another hospital and was treated as a case of pneumonia where she received antibiotic but with no improvement. Computerized tomography (CT) chest showed diffuse interlobular septal thickening in the background of ground glass opacity giving a picture of crazy paving pattern which was consistent with the diagnosis of PAP. The patient was scheduled to undergo, first right-sided whole lung lavage (WLL) under general anesthesia. Endobronchial intubation using left sided 37 Fr double lumen tube. Continuous positive airway pressure (CPAP) as described in our previously published report was connected to the right lumen of the endobronchial tube. CPAP ventilation was used during the suctioning of lavage fluid phase in order to improve oxygenation. WLL was done using 5 L of warm heparinized saline (500 i.u/litre). The same procedure was repeated on the left side using 6 L of heparinized normal saline solution. In conclusion, anesthesia in alveolar proteinosis for patients undergoing WLL is challenging to the anesthesiologist. It requires meticulous preoperative preparation with antibiotics, mucolytics and chest physiotherapy. Also it requires careful intraoperative monitoring and proper oxygenation especially during the suctioning phase of the lavaged fluid. With this second case report of successful anesthetic management using the modified CPAP system we recommend with confidence the application of CPAP ventilation to improve oxygenation during WLL.

## INTRODUCTION

Pulmonary alveolar proteinosis (PAP) is a rare disorder characterized by accumulation of lipoproteinacious substance in the bronchoalveolar tree.[1] The disease was first coined by Rosen in 1958.[2] Recent studies showed that the administration of human granulocyte-macrophage colony-stimulating factor (GM-CSF) is effective treatment of PAP.[3] In spite of that whole lung lavage (WLL) remains the most effective treatment of PAP[4]. WLL, under general anesthesia, is challenging due to the difficulty in maintaining oxygenation during the procedure. Different methods have been suggested to deal with hypoxia during WLL. In a previous report we have described a novel technique using modified continuous positive airway pressure (CPAP) system to improve oxygenation during WLL[5]. In this report we describe another case of PAP but with different clinical presentation, where we used the same CPAP technique during WLL.

## CASE REPORT

**Figure 1:**
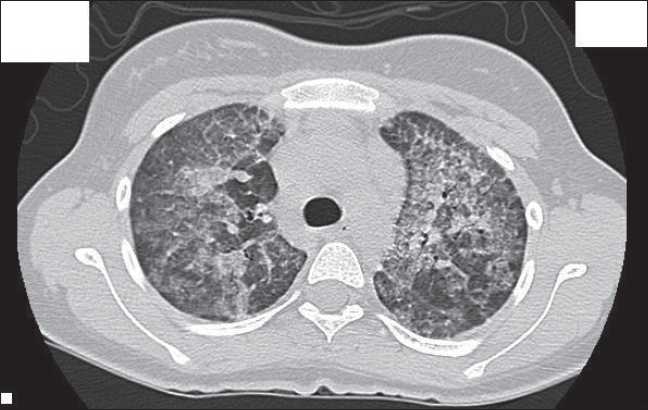
CT lung scan showing crazy paving pattern

A 15-year-old female who had history of cough with sputum for 3 days along with fever was presented to us. She was seen in another hospital and was treated as a case of pneumonia where she received antibiotic but with no improvement. She was still having shortness of breath, so H1N1 was suspected; however, she was investigated with negative findings. Her previous medical history showed that she had chronic history of cough, easy fatigue-ability and shortness of breath on mild exertion. Her family history showed that her sister died from chronic respiratory disease, and also her two brothers have similar symptoms and they suffered from chronic lung disease. Further investigations were done including CT chest which showed diffuse interlobular septal thickening in the background of ground glass opacity giving a picture of crazy paving pattern with multiple patchy air space consolidation more on the lower lobes with air bronchogram [Figure 1]. After that the patient was sent for lung biopsy. Open lung biopsy done, which confirmed PAP. The patient was transferred to our hospital for WLL. Upon receiving she was conscious on oxygen (oxygen saturation 97% on 2-3 L of O_2_), pulse rate 96 beats/min, blood pressure 115/60 mmHg, respiratory rate was 24/min and body temperature was 36.8ºC. Biochemical analysis data were all within normal ranges. Arterial blood gases on room air were: PaO_2_72.4 mmHg, PaCO_2_ 34.9 mmHg, pH 7.426 and HCO_3_ 2.4 mmol/L. Pulmonary function tests (PFTs) done and showed restrictive pattern.

**Figure 2:**
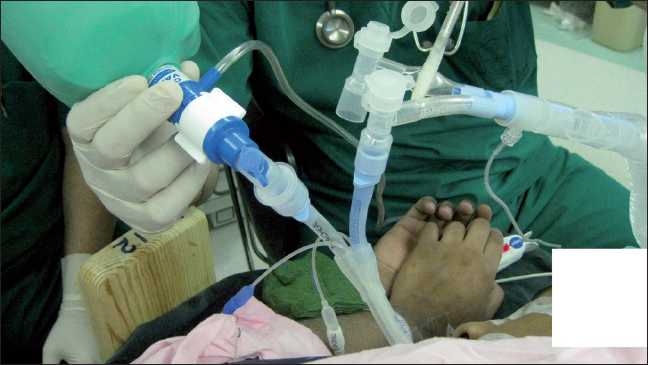
CPAP bag connected to the lumen of DLT going to the lavaged lung

Since the right lung was mostly affected, the patient was scheduled to undergo, first right-sided WLL under general anesthesia. Premedication was achieved with 1 mg oral lorazepam. After connecting the patient to standard monitoring, induction of anesthesia was achieved with 100 mcg fentanyl and 200 mg propofol i.v. and endobronchial intubation using left-sided 37 Fr double lumen tube (Silbronch, Fuji, Japan) was facilitated with 8 mg cisatracurium i.v. The patient was placed in right lateral position with mostly the right side down. Right lung was isolated using a clamp placed on the right lumen of the endobronchial tube. CPAP as described in our previously published report was connected to the right lumen of the endobronchial tube [Figure 2].[5] CPAP ventilation was used during the suctioning of lavage fluid phase in order to avoid the accompanying desaturation. WLL was done using 5 L of warm heparinized saline (500 i.u/L). Following the procedure, the trachea was extubated and the patient was transferred to the high dependency unit (HDU) for further observation. Her postoperative recovery was uneventful. One day later she was transferred back to the ward.

**Figure 3:**
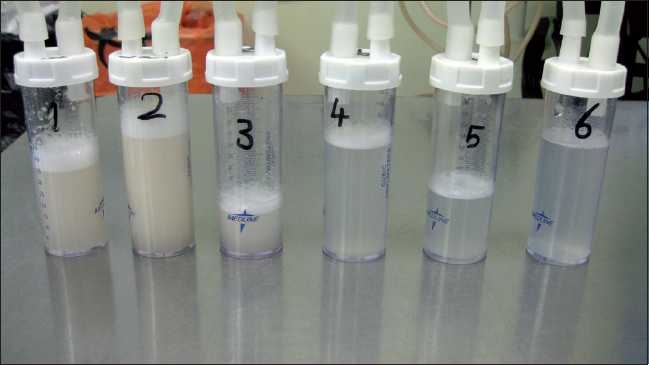
Effluent fluid became clearer

The patient again was scheduled after 1 week for left-sided WLL where the same anesthesia and ventilation techniques were used. This time 6 L of warm heparinized saline were used until the effluent became clear [Figure 3]. The patient was extubated and shifted to HDU. Her postoperative period was smooth and eventually she was shifted to the ward on the next day. The patient did well after the procedure, maintaining saturation 98% oxygen on room air. Postoperative PFTs were done and she showed slight improvement. The patient was discharged in good condition to be seen in the clinic after 1 month.

## DISCUSSION

PAP is a rare disease that affects young population usually in the age group of 20-40 years, characterized by the deposition of lipoproteinacious material in the alveoli secondary to abnormal processing of surfactant by macrophages.[4] PAP also occurs in infancy, resulting from abnormalities of surfactant production or decreased catabolism of surfactant where the only effective treatment is bronchoalveolar lavage.[6] WLL for treatment of PAP consists of two phases-instillation and suctioning. Severe hypoxemia is often accompanying the suction of lavage fluid phase during WLL. Therefore, several methods have been suggested to overcome this problem. In one study it was found that during lung lavage for PAP, hyperoxygenated solution has significantly improved oxygenation during the procedure.[7] In the present case we have used a simpler method to improve oxygenation during WLL represented by the so-called modified CPAP system which we have adopted several years ago.[5] In one case report of severe PAP, the patient underwent WLL and required the use of extracorporeal membrane oxygenation to improve oxygen supply.[8] In a similar case, high-frequency jet ventilation through a fiberoptic bronchoscope channel was used for the same purpose.[9] In WLL, we start with the worst side of the lung-in our case it was the right lung. The lung to be lavaged should be the down-most side and this to avoid any kind of artificial cardiac tamponade secondary to the pressure on myocardium which can be exerted during the instillation of normal saline phase of WLL. Usually during the instillation phase oxygenation was maintained. In contrary, during the suctioning phase of the lavaged fluid desaturation occurs which necessitates oxygen supplementation.

In conclusion, anesthesia for WLL in alveolar proteinosis is challenging to the anesthesiologist. It requires meticulous preoperative preparation with antibiotics, mucolytics and chest physiotherapy. Moreover it requires careful intraoperative monitoring and proper oxygenation especially during the suctioning phase of the lavaged fluid. With this second case report of successful anesthetic management using the modified CPAP system we recommend with confidence the application of CPAP ventilation to improve oxygenation during WLL.
